# Fascial Manipulation Compared to Stretching for Rehabilitation of Children With Cerebral Palsy: Protocol for a Pilot Randomized Controlled Trial

**DOI:** 10.2196/92401

**Published:** 2026-09-30

**Authors:** Karin von Smitten-Stubb, Joachim Ring, Piia Haakana, Heikki Hurri, Mira Huusko, Ira Jeglinsky-Kankainen, Jyrki Kettunen, Juha-Pekka Kulmala, Taru Laakso, Tiina Lahtinen-Suopanki, Essi Marttinen Rossi, Helena Mäenpää, Tuula Niemelä, Mika Peltoniemi, Leena Ristolainen, Petri Salonen, Eva Teijonsalo, Nita Tolvanen

**Affiliations:** 1 University of Helsinki, Helsinki, Uusimaa, Finland; 2 Arcada University of Applied Sciences, Helsinki, Uusimaa, Finland; 3 Helsinki Children's Hospital, Stenbäckinkatu 9, Helsinki, Uusimaa, Finland, 358 40 4863595; 4 Research Institute Orton, Helsinki, Finland; 5 Finnish Education Evaluation Centre, Helsinki, Finland; 6 Terapia-asema Voima Oy, Espoo, Finland; 7 Independent physiotherapist, Helsinki, Finland; 8 Motion Analysis Laboratory, Helsinki University Hospital, Helsinki, Finland; 9 University of Helsinki, Children and Adolescent, Helsinki, Finland

**Keywords:** fascial manipulation, cerebral palsy rehabilitation, stretching, walking, spasticity, range of motion

## Abstract

**Background:**

Maintaining and improving motor function is essential in the rehabilitation of children with cerebral palsy (CP). Stretching is traditionally used to prevent muscle tightness and contractures to improve movement patterns in children with CP. Fascial manipulation (FM) is an increasingly used manual therapy method for movement difficulties associated with CP. However, evidence for FM remains limited, and its benefits over conventional stretching are unclear. A pilot randomized controlled trial (RCT) is warranted to assess the feasibility and acceptability of a definitive trial comparing FM with stretching. This study protocol describes a pilot randomized controlled feasibility trial comparing FM and stretching in children with CP to inform the design and conduct of a future definitive RCT.

**Objective:**

The primary objective is to assess the feasibility and acceptability of the trial procedures and interventions. This includes evaluating recruitment and retention rates, intervention adherence, completion rates for outcome assessments, and the perceived impact of the interventions on children’s daily functioning and participation. The secondary objective is to explore the preliminary acute and longitudinal effects of FM, compared with traditional stretching, on walking performance, gait biomechanics, joint mobility, and the variability in these outcome measures.

**Methods:**

This pilot RCT will include 20 children aged 7 to 14 years with spastic CP, randomized to an FM intervention group (n=10) and a stretching group (n=10). Both groups will receive 1 treatment session per week for 8 weeks. Feasibility and acceptability will be assessed using quantitative measures of recruitment and retention, intervention adherence, and completion of outcome assessments, complemented by semistructured interviews with caregivers and the children’s regular physiotherapists to explore perceived changes in the children’s daily functioning and participation, and their experiences of the interventions. Secondary outcomes include the 6-minute walk test (6MWT), walking metabolic cost, gait biomechanics, ankle spasticity, muscle activity, joint stiffness, and functional mobility in daily life. Acute outcomes will be assessed before and after the first intervention session, while longitudinal outcomes will be assessed at baseline and following the 8-week intervention period.

**Results:**

Recruitment of participants began in October 2024, with 3 additional participants required to reach the target sample size. Results are expected to be published in 2027.

**Conclusions:**

This pilot RCT will provide essential feasibility data and preliminary estimates of potential treatment effects to inform the design of a future adequately powered RCT. To our knowledge, evidence regarding the effects of FM on gait, mobility, and function in children with CP remains limited. This study aims to contribute to the development of evidence-based rehabilitation strategies for children with CP.

## Introduction

### Background

Cerebral palsy (CP), a condition caused by damage to the brain’s structure and function, is the most common and severe movement disorder in children, with a prevalence of 1.6 per 1000 live births [1,2]. The brain injury itself is permanent and nonprogressive, but its consequences change over time [3]. CP is an umbrella term for a group of movement and functional disorders that encompass a highly heterogeneous population with varying levels of motor ability and functional capacity [3]. Therefore, CP is classified into subtypes based on clinical localization, predominant movement patterns, and functional capacity. The most common subtype is spastic CP, which accounts for approximately 80% of all CP cases [4].

For the classification of function in children with CP aged 0 to 18 years, clinicians and researchers usually use the Gross Motor Function Classification Scale (GMFCS) and Gross Motor Function Classification System—Expanded and Revised (GMFCS-ER). The GMFCS describes gross motor function, limitations of everyday activities, functional performance, and the use of assistive devices [5]. The classification is rated from I to V, with level I indicating minimal impairment of gross motor function [5].

### Spasticity and Contractures

CP resulting from upper motor neuron damage causes a sensorimotor disorder characterized by a velocity-dependent increase in muscle tone and stretch reflex activity [6]. Furthermore, it can show nonvelocity-dependent involuntary background activation. Nonneural factors (specifically, altered muscle mechanical properties) are also frequently present and limit joint range of motion (ROM) [7].

Although both neural and nonneural factors contribute to the joint hyper-resistance and movement difficulties observed in children with CP [8], nonneural factors may also play key roles in maintaining long-term functional ability [9,10]. This is because alterations in muscle mechanical properties (eg, increased stiffness) significantly impair a muscle’s ability to lengthen. Consequently, although the early brain injury in CP is nonprogressive, the associated musculoskeletal problems tend to worsen over time [11,12]. Impaired muscle lengthening capacity, especially during growth spurts, can rapidly reduce joint ROM, potentially leading to permanent muscle shortening. This condition, known as contracture, is characterized by abnormally stretched sarcomeres, a reduced number of sarcomeres in series, and alterations in collagen and the extracellular matrix (ECM) [13]. Children with CP can, as they grow, develop a permanent shortening of their musculotendinous units [14]. Contractures have clinically significant consequences, as they are associated with joint and bone deformities, pain, and loss of mobility [15].

### Gait in CP

The combination of muscle weakness, reduced ROM, contractures, and joint hyper-resistance often leads to altered gait patterns in children with CP [7,16]. These changes can result in a decrease in, or even the loss of, walking ability, while also causing difficulties in standing, balance, mobility, and overall functional capacity [16]. Walking ability in individuals with CP varies significantly depending on the severity of the motor deficits [17].

A common movement deviation in CP is crouch gait, which arises from short and spastic hamstring muscles. It is characterized by excessive knee and hip flexion and is often accompanied by in-toeing or out-toeing [18]. Another frequently seen deviation is toe-walking, which is associated with ankle equinus (increased ankle plantarflexion) resulting from short and spastic ankle plantar flexors [19]. These and other alterations in gait patterns can severely impair walking efficiency in children with CP [18], leading to increased energy consumption and fatigue during walking compared to healthy peers [20]. Thus, one of the main goals of rehabilitation for children and youth with GMFCS levels I to III is to improve walking ability, as enhanced walking can support the development of other motor functions while also promoting greater participation in daily life activities [17].

### Stretching

Both active and passive stretching have traditionally been used in rehabilitation to prevent muscle tightness and contractures and to improve movement patterns in children with CP [21,22]. The effects of stretching have been well studied; acute and long-term results show an acute increase in ROM of around 10° [22,23]. However, Harvey et al [24] found that stretching does not have a clinical impact on ROM. Kalkman et al [22] found that the muscles and tendons in children with CP do not exhibit acute changes following a series of stretching sessions, suggesting that they do not contribute to the observed increase in ROM. Instead, there was an increase in the maximal ankle joint torque tolerated by the participants [25].

Various factors, including alterations in muscle length [26], tendon length [22,27], and cross-sectional area [28], as well as changes in connective tissue properties [27], may contribute to muscle stiffness and reduced flexibility in CP. Furthermore, it has been suggested that the tendons may be longer and more compliant in children with CP than in their typically developing peers.

Indeed, in ankle plantar flexors, stretching exercises have been shown to primarily elongate the more compliant Achilles tendon rather than the muscle itself [29]. In children with CP, an increased amount of connective tissue within the muscle is associated with a reduced ability of the muscle to extend [23]. The lengthening of the muscle belly depends on the properties of both the muscle fascicles and the surrounding connective tissues—specifically the perimysium and endomysium [22]. As a result, fascial tissue may be the primary limiting factor in muscle elongation and should, therefore, be the main target during static stretching [30,31]. Although acute increases in ROM have been observed following stretching, there is no clear evidence supporting its effectiveness in preventing contractures [24]. Consequently, treatment strategies that more effectively target muscle connective tissues, such as fascia, could potentially lead to better outcomes in increasing ROM.

### Fascial Manipulation

The role of fascial treatments in the rehabilitation of children with CP has received only limited attention. Fascial manipulation (FM) is a manual treatment that takes a different approach, targeting the deep fascia rather than focusing on the muscle fascicles, as in more frequently used stretching. Interventions targeting the deep fascia, particularly procedures scheduled before the onset of contractures and deformities, may be an essential strategy for improving motor function in this population.

The fascia consists of collagen fibers, loose connective tissue, and hyaluronic acid (HA) [32]. HA is a polysaccharide in the ECM of connective tissues [33]. In muscle tissue, HA functions as a lubricant within the endomysium, perimysium, and epimysium to facilitate the sliding of muscle fibers during movement [32]. Variations in the viscosity of HA within the fascia occur with mechanical loading and influence the fascia’s mechanical behavior and structure. During loading, viscoelasticity is reduced, and during rest, HA regains a viscous state [32]. The capsules of the muscle spindles that sense muscle stretch are connected to the perimysium, attach in multiple directions, and extend into the deep muscular fascia. This suggests that fascial tension may influence spindle sensitivity and contribute to increased muscle hyper-resistance [10,34-36]. Therefore, modulating fascial tension may represent a feasible and promising approach to maintaining the functional integrity of the muscle spindles to modulate the peripheral component of spasticity [35,36].

A trademarked manual therapy method for this type of fascial tension modulation is the Stecco FM protocol developed by physiotherapist Luigi Stecco [37-39]. FM relies on deep friction to restore gliding of the tissues of the deep fascia, aiming to influence the hyaluronan within the fascia connected to the muscle fibers [38,39]. Emerging evidence has shown increased joint ROM in the upper limb following FM, possibly due to decreased viscosity of the ECM [39,40]. Treatment points are selected based on a thorough assessment, which includes the patient’s medical history, movement analysis, and palpation tests [41]. For the treatment of spasticity, FM is directed to the spastic body parts to target the deep fascia associated with muscles, including the aponeurotic and epimysial fascia.

In children with CP, FM has emerged as a promising treatment for the upper limbs, as it reduces spasticity, increases ROM, and improves muscle strength [42]. Additionally, hyaluronidase injections applied to spastic upper extremities in adult patients, with injection points chosen based on clinical reasoning determined by FM, have shown positive effects on both active and passive movement [34]. This study also supported the application of this treatment to more segments than only the most spastic ones [34]. Notably, FM has demonstrated similar effects [38-40]. A qualitative interview study [43] conducted in Finland demonstrated that the inclusion of FM within neurological physiotherapy facilitated active movement and increased passive ROM for children with CP [43].

Although studies on FM are increasing, the evidence favoring its use remains limited, with pain frequently used as the primary outcome measure [36]. Despite clinical experience supporting the use of FM in the rehabilitation of children with CP, the lack of evidence, particularly regarding its effects on the lower extremities, highlights the need for further investigation into the potential of FM as an effective intervention to increase ROM and reduce spasticity, thereby enhancing physical function in this population.

### Study Purpose

#### Primary and Secondary Aims

The primary aim of this pilot randomized controlled trial (RCT) is to evaluate the feasibility and acceptability of conducting a future definitive RCT comparing FM with traditional stretching in children with CP. Specifically, the study will evaluate the feasibility of participant recruitment, retention, intervention delivery, adherence, outcome assessments, and data collection procedures. Semistructured interviews with parents and the children’s regular physiotherapists will provide qualitative data on the feasibility and acceptability of the interventions and their perceived impact on the child’s daily functioning and participation in daily activities [44].

The secondary aim is to explore the preliminary acute effects (following the first treatment session) and longitudinal effects (following the 8-wk intervention) of FM, compared with traditional stretching, on walking performance, gait biomechanics, and joint mobility, as well as variability in these outcome measures. Exploratory outcomes include the 6-minute walk test (6MWT), walking efficiency, gait pattern, joint ROM, ankle joint stiffness, and spasticity.

As this is a pilot RCT, the study is not powered to detect statistically significant between-group differences; instead, the clinical outcomes will be used to estimate outcome variability and preliminary treatment effects for planning a future definitive RCT.

#### Hypotheses

The primary hypothesis is that the trial procedures and intervention will be feasible and acceptable, with adequate recruitment, retention, intervention adherence, and completion of outcome assessments to inform the design of a future definitive RCT.

The secondary hypothesis is that FM results in greater preliminary improvements in walking performance, gait biomechanics, and joint mobility than traditional stretching, both acutely and following the 8-week intervention.

## Methods

### Study Design

This study is a 2-arm pilot RCT with an 8-week intervention period and a convergent mixed methods design. Quantitative data will be collected through preintervention, acute response, and postintervention assessments, while qualitative data will be collected through semistructured interviews conducted after completion of each child’s intervention [45]. The quantitative and qualitative findings will be integrated during data interpretation to provide complementary information regarding the feasibility and acceptability of the study procedures and the preliminary effects of the interventions [46].

Participants will be randomly allocated in a 1:1 ratio to 1 of 2 intervention groups. One group will receive targeted FM once weekly for 8 weeks, while the comparison group will receive traditional stretching once weekly for the same period (Figure 1). The schedule of enrollment, interventions, and assessments is presented in Table 1.

This study is conducted in collaboration among Orton Hospital (hereafter, Orton), the New Children’s Hospital, HUS Helsinki University Hospital, and Arcada University of Applied Sciences. Recruitment began in October 2024, and the study is expected to be completed in December 2027.

The study design is convergent, meaning that the interventions, assessments, and qualitative interview data are collected, analyzed, and reported simultaneously [47].

**Figure 1.**
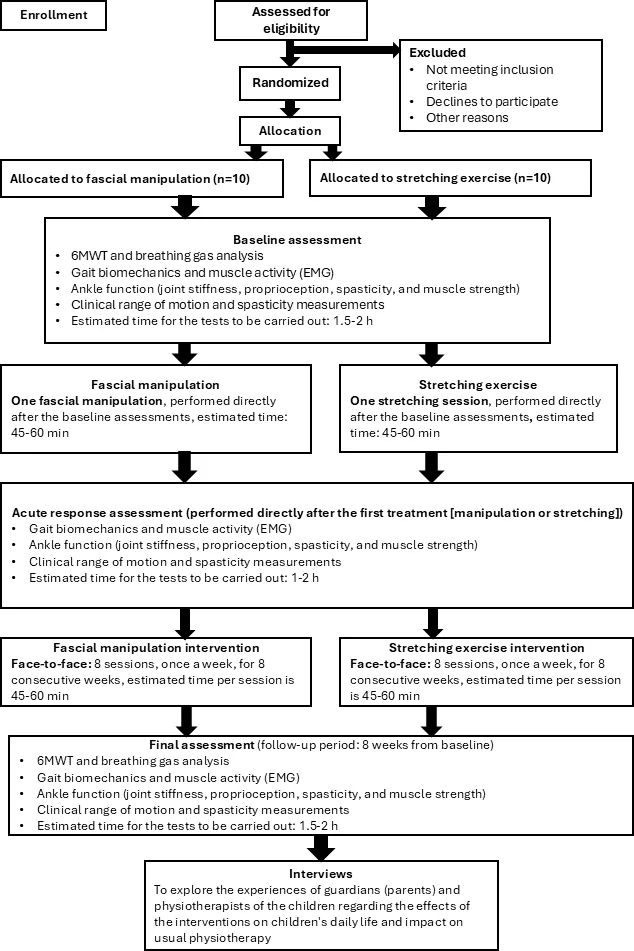
Flowchart of the study protocol. EMG: electromyography; 6MWT: 6-minute walk test.

**Table 1.** Schedule of enrollment, interventions, and assessments.

Study procedures	Study period and time point								
	Enrollment	Baseline assessments	First intervention	Acute response assessments		Intervention period^a^^,b^	After assessment		Close-out
	–t_1_	t_0_		t_1(directly)_	t_2(1 h after)_		t_3 (8 wk)_	t_4_	t_x_
Enrollment									
Eligibility screen	✓								
Informed consent	✓								
Random allocation	✓								
Interventions									
Fascial manipulation			✓			**✓** ^a^			
Stretching exercises			✓			✓^b^			
Assessments									
Clinical tests		✓			✓		✓		
6MWT^c^		✓					✓		
Walking metabolic cost		✓					✓		
Ankle joint hyper-resistance		✓		✓	✓		✓		
Gait pattern		✓			✓		✓		
Interview								✓	
Compliance to intervention				✓	✓	✓	✓	✓	✓
Acceptability of intervention									✓

a Fascial manipulation (8 times).

b Stretching exercise (8 times).

c 6MWT: 6-minute walk test.

### Study Population

Twenty children fulfilling the eligibility criteria shown in Textbox 1 will be included.

Textbox 1. **Inclusion and exclusion criteria.**

**Inclusion criteria**

- Children aged 7 to 14 years with spastic cerebral palsy
- Gross Motor Function Classification Scale levels II and III
- In addition to age and Gross Motor Function Classification Scale level, inclusion for recruitment is that the child can cooperate in measurement and intervention
- Family living in the Helsinki metropolitan area

**Exclusion criteria**

- The child has previously received fascial manipulation administered by a Stecco-trained therapist or other fascia treatment during the last 6 months
- Surgery in the lower extremities during the last 12 months
- Botox injection in the lower extremities during the last 6 months
- Muscle-electrical stimulation during the last 3 months
- Reduced cognitive level
- Severe visual impairment (unable to perform the walking test)
- Other illness: psychological or other serious illness

### Sample Size

This pilot RCT is designed to assess the feasibility and acceptability of the trial procedures and interventions and to inform the design of a future definitive RCT. The sample size was therefore determined based on feasibility considerations rather than on statistical power to detect treatment effects. A total sample of 20 participants (10 per group) was considered appropriate to assess key feasibility outcomes, including recruitment and retention, intervention adherence, and completion of outcome assessments, while also providing preliminary estimates of outcome variability to inform the planning of a future definitive trial. Consistent with recommendations for pilot and feasibility trials, the sample size was justified according to the objectives and feasibility outcomes of the pilot rather than on formal hypothesis testing of intervention effects [48,49]. Secondary clinical outcomes will be considered exploratory and will not be used to determine the sample size.

### Recruitment Procedure

Participants are being recruited through the patient register of the Helsinki University Hospital New Children’s Hospital (HUS/ULS). A research coordinator involved in the project at HUS/ULS initially selects from the register those children who meet the inclusion criteria. Subsequently, once the participants are identified and contacted, an information letter is sent to their families regarding their preliminary willingness to participate in the study. The letter provides information about the study, its purpose, and what participation in the project entails for the participants and their families. Upon expression of interest, participants will be screened against the eligibility criteria, and the participant information sheet and consent form will be sent to prospective participants via email. If the participant or guardian refuses, a new participant will be identified from the register. The recruitment process, which started between August and September 2024, will end when the required numbers of participants in both groups are reached.

### Randomization and Allocation

If both the participant and the guardian preliminarily agree to participate, the distribution to intervention groups is conducted using a computer-based randomization tool. The primary randomization strategy is permuted block randomization with block sizes of 4, stratified by age, sex, and GMFCS level. To account for potential recruitment constraints in this population, a matched-pair allocation procedure is prespecified as a fallback strategy if the primary block randomization procedure cannot be maintained for the remaining participants. Under this procedure, participants will be prospectively matched in pairs based on GMFCS level, age, and sex. Once a matched pair has been formed, the 2 participants will be randomly allocated in a 1:1 ratio to either the FM group or stretching group. This process will be repeated until both intervention groups are filled. The child and the guardian are not informed about their group assignment until they have provided their agreement to participate.

The nature of the intervention precludes blinding of either participants or staff to allocation. However, 2 members of the research team and the study statistician will remain blinded to group allocation during data preparation and statistical analysis to minimize the risk of bias in data interpretation. In the flowchart (Figure 1), the timeline of the study is visualized in relation to the progress of the actual RCT, and the flowchart’s functionality is being evaluated in this pilot.

### FM Intervention

In this research protocol, FM will be performed once a week for 8 weeks at home or at school by a therapist highly educated in the FM method. The aim of the manual friction treatment of specific points in the deep fascia is to increase the local temperature, as this can reverse the aggregation of HA, thereby restoring the gliding of the underlying fascial layers, enhancing the function of muscle-related deep fascia, enabling movement, and allowing overstrained or painful structures to heal [40,41,50].

The treatment points in FM are selected by a specific protocol that includes clinical history-taking, an assessment of movement and palpation, which directs an individual choice of treatment [38,39]. A specific structured assessment approach is used, with emphasis on chronology, previous musculoskeletal events, and patient symptomatology [39]. Palpatory verification of the deep fascia of the most affected segments defines which centers of coordination points, centers of fusion points, and myofascial chains are treated [39]. Treatment of muscles located both proximally and distally to the primary stiffness along the agonist and antagonist myofascial chains gives better results [34]. Excessively painful areas are initially excluded to enhance patient comfort and therapeutic effectiveness [51]. The same functional movement that is assessed before treatment is assessed again afterward to actively engage with the functional changes and provide experience with altered muscle function.

### Stretching Intervention

Passive stretching is performed once a week, supervised by a physiotherapist, in the participant’s home or school. The stretching protocol follows the procedure described by Valadão et al [52], with the addition of stretching the calf muscles [52]. The muscles are stretched by moving the joint to the end-range position. Four sets of 45 seconds of manual passive-static stretches at the pain threshold (ie, the position at which the participant acknowledges an initial pain sensation) are performed for each muscle group. The stretched muscles are the 1- and 2-joint hip flexors, hip adductors, knee flexors, and calf muscles. A Tabata timer on a cellular phone is used to standardize the duration of the stretches. Table 2 shows the protocol for passive stretching. This stretching protocol targets the hip, knee, and ankle, the commonly affected joints of contractures in children with CP [15].

Regular physiotherapy for participants in both groups continues as scheduled. The physiotherapist responsible for each participant’s usual physiotherapy receives information about the intervention study (eg, by telephone) to coordinate the participant’s usual physiotherapy with participation in the intervention study. This arrangement is applied for ethical reasons and is intended to enable a more reliable assessment of the potential added value of including FM in rehabilitation.

**Table 2.** Stretching is conducted once per week in the child’s home or school by a physiotherapist using a Tabata timer app to standardize the timing. All stretches are passive-static and applied at the pain threshold (ie, at the onset of initial stretch discomfort).

Muscle group	Position	Technique details	Stretching duration	Break duration
One- and two-joint hip flexors	Modified Thomas test (supine on table)	One leg held in full hip flexion (assisted, if needed); the other leg hangs off the table in extension. Torque is applied by the physiotherapist at the distal thigh. Knee either fully flexed or relaxed.	4 × 45 s per leg	45 s between sides
Hip adductors	Seated upright with straight legs in adduction	Physiotherapist maintains leg alignment to prevent rotation. Torque is applied at the posterior shank by the physiotherapist. Both legs stretched simultaneously.	4 × 45 s (both legs simultaneously)	None
Hamstrings	Supine, leg hip flexed to ~90°	Unstretched leg secured with hip ~0°. Extension of knee on stretched leg assisted by physiotherapist.	4 × 45 s per leg	45 s between sides
Soleus	Prone lying with knee at 90°	Torque applied in ankle dorsiflexion by physiotherapist.	4 × 45 s per leg	45 s between sides
Gastrocnemius	Standing, leaning toward wall with stretched leg extended	Wall stretch posture and assistance, if needed.	4 × 45 s per leg	45 s between sides

### Interviews

Data collection will include semistructured interviews with the participant’s guardian (parent) and the children’s regular physiotherapists in both groups after the final assessment. The number of parent interviews will equal the number of child participants: 10 parents of children who participated in the FM group and 10 parents of children who participated in the stretching group. If the child attends physiotherapy regularly, their physiotherapist will be asked to participate in an interview, resulting in interviews with approximately 20 physiotherapists.

The researcher will contact each interviewee to arrange a suitable interview time. The interview will be conducted either via Microsoft Teams (remote connection) or face-to-face. Each interview will last approximately 30 minutes. The interviews will be recorded with the interviewees’ consent.

### Outcome Measures and Assessment Methods

#### Primary Feasibility Outcomes

##### Primary Outcome Assessments

The primary outcomes of this pilot RCT are the feasibility, acceptability, and safety of the trial procedures and interventions, with the aim of informing a future definitive RCT comparing FM with traditional stretching in children with CP. Feasibility will be assessed across participant recruitment and retention, intervention delivery and adherence, outcome assessment, and data collection. Acceptability will be explored through semistructured interviews with parents and the children’s regular physiotherapists, focusing on their experiences of the interventions and study procedures, the perceived burden of participation, and the perceived impact on the children’s daily functioning and participation.

##### Recruitment and Retention

The numbers of participants screened, eligible, recruited, and retained during the 8-week intervention will be documented, together with recruitment and retention rates.

##### Intervention Delivery and Adherence

Intervention delivery will be assessed by the proportion of planned intervention sessions successfully delivered, while adherence will be assessed by the proportion of prescribed sessions completed. Reasons for missed, discontinued, or undelivered sessions will be documented, where applicable.

##### Outcome Assessment and Data Collection

Feasibility will be assessed based on completion rates of planned assessments and the availability of data for predefined outcome measures at each assessment time point. Reasons for missing data or incomplete assessments will be recorded where applicable.

##### Acceptability

Acceptability will be explored through semistructured interviews with parents and children’s regular physiotherapists, addressing experiences with the interventions and study procedures, perceived burden of participation, and perceived effects on daily functioning and participation.

##### Safety

The frequency, severity, and potential relatedness of adverse events (AEs) and serious adverse events (SAEs) to the study interventions will be recorded throughout the intervention and follow-up periods.

##### Feasibility Assessment

Feasibility outcomes will be evaluated descriptively across the key domains of recruitment, retention, intervention delivery and adherence, outcome assessment, and data collection. Recruitment and retention rates, intervention adherence, and completion rates for planned outcome assessments will be reported with corresponding 95% CIs, where appropriate. These feasibility outcomes will be interpreted collectively, together with qualitative information on acceptability and practical barriers to participation and intervention delivery, to assess the practicality of conducting a future definitive RCT and to identify potential modifications to the study procedures.

In addition, basic descriptive information will be collected from participants and their parents, including age, sex, CP subtype and GMFCS level, other health conditions, previous surgical treatments, current medications, and ongoing rehabilitation.

### Secondary Outcomes

#### Secondary Outcome Assessments

Participants will attend the Movement Analysis Laboratory at the New Children’s Hospital (HUS/ULS) for testing sessions. They will undergo a series of assessments, including the 6MWT, walking metabolic cost, 3D gait analysis, ankle hyper-resistance testing using an isokinetic dynamometer, and clinical tests, at baseline, receive one FM treatment, and then undergo repeated assessments 1 hour after treatment (except for the 6MWT) to assess the acute response to the therapy. The 6MWT is excluded from the acute response assessments conducted after the therapy session, as its inclusion would make the overall duration and physical demand of the session too strenuous. Ankle hyper-resistance is also measured immediately after the treatment. After 8 weeks (about 2 mo) of treatment, the participants will undergo the same measurements again to assess the longitudinal effect of the therapy.

#### Walking Capacity

The 6MWT will be used to assess changes in walking capacity. Since the interventions in this study aim to reduce joint hyper-resistance in children with CP, an improvement in walking capacity can be expected. The 6MWT will be carried out in a corridor with a 30 m test track. The course has lines at 5 m intervals to simplify calculation of the walking distance. The outcome of the 6MWT is the distance walked during the 6 minutes of the test and is noted with 1 m accuracy. The 6MWT is a reliable tool for assessing changes in walking capacity following various interventions aimed at improving motor function in this population [53].

#### Walking Metabolic Cost

Measurement of walking metabolic cost gives an opportunity to assess and understand how much effort a participant expends to achieve the result in the 6MWT. A lower energy consumption after an intervention would indicate more economical walking, even if walking distance does not change. Metabolic cost is determined by calculating the energy cost of walking using the following formula: Energy cost (J/kg/m) = (4.960 × respiratory exchange ratio + 16.040) × VO_2_ (mL/kg/min) / walking speed (m/min). The respiratory exchange ratio is calculated by dividing VO_2_ by VCO_2_. These values represent the average oxygen uptake and carbon dioxide production during the last 2 minutes of the 6MWT, respectively, relative to body weight (mL/kg/min). The Cosmed K5 (COSMED Srl) mobile metabolic system is used to collect VO_2_ and VCO_2_ data in this study [54].

#### Gait Pattern

Changes in gait pattern will be determined during walking using the Gait Deviation Index (GDI) derived from 3D motion analysis. Measurements will be performed in a gait laboratory using an 18-camera Vicon motion-capture system. A GDI (scale 0‐100) score of 100 indicates a gait pattern similar to the normative data, while lower scores indicate greater deviations from normal gait. Every 10 points represents 1 SD away from the normative mean [55].

#### Ankle Joint Hyper-Resistance and Strength

Ankle joint stiffness and the triceps surae muscle response to stretching will be determined using an ankle test protocol on an isokinetic dynamometer (Con-Trex), together with electromyography recordings. The passive (ie, muscle stiffness) and neural (ie, spasticity) components of joint hyper-resistance will be separated by conducting ankle dorsiflexion movements at slow (10°/s) and fast speeds (up to 300°/s) under relaxed conditions [56]. The appropriate ROM for the foot attachment will be determined during the pretest by moving the ankle toward dorsiflexion until the passive torque reaches 10 to 15 Nm [57]. The same ROM and dimensional settings will be used during the posttest to enable pre-post comparisons. The resulting increase in passive joint moment and reflex response (soleus and medial gastrocnemius) will then be analyzed under both slow and fast conditions. Measurements at low speed determine passive ankle joint stiffness, while measurements at fast speed represent stretch hyperreflexia (or spasticity). In addition, maximal voluntary dorsiflexion and plantarflexion strength will be measured for each participant. In the dynamometer test, the patient will be seated with a backrest angle of 70°, with the thigh strapped to the seat, the knee extended, and the foot secured to the manufacturer’s standard footplate attachment. Muscle activity will be recorded from the soleus, medial gastrocnemius, and tibialis anterior muscles.

#### Clinical Tests

Clinical assessments will include active and passive joint ROM, spasticity, and selective motor control of the lower extremity. Active and passive ROM will be assessed to characterize changes in joint mobility and voluntary movement capacity [58]. Spasticity will be evaluated using the Modified Tardieu Scale (MTS), which assesses the muscle response to passive movement performed at slow and fast velocities and provides information on the dynamic component of increased muscle tone [59]. Selective motor control will be assessed using the Selective Control Assessment of the Lower Extremity (SCALE), which evaluates the ability to perform isolated voluntary movements of the lower extremity [60]. These clinical assessments will complement the instrumented measures of ankle joint hyper-resistance and gait biomechanics by providing clinically relevant information on joint mobility, spasticity, and motor control.

### Data Management and Analysis

As a feasibility pilot RCT, the statistical analysis will focus primarily on descriptive statistics to evaluate trial feasibility, acceptability, and safety. Recruitment, eligibility, retention, and intervention adherence rates will be calculated with corresponding 95% CIs.

The exploratory clinical outcomes will be analyzed separately according to the timing of outcome assessment and the specific research question. Acute outcomes measured at baseline and immediately following the first treatment session will be analyzed using linear mixed-effects models (LMMs), with treatment group, time, and their interaction specified as fixed effects, and a random intercept for individual participants to account for within-participant correlations. For acute outcomes measured at 3 time points (baseline, immediately posttreatment, and 1 h posttreatment), the same LMM framework will be applied with time specified as a 3-level categorical fixed effect to characterize the immediate treatment response and its temporal evolution during the first hour following treatment. Longitudinal outcomes measured at baseline and after the 8-week intervention will be analyzed using a corresponding LMM framework, with treatment group, time, and their interaction specified as fixed effects and participant specified as a random intercept. For outcomes measured at both acute and longitudinal time points, separate analyses will be conducted to address the acute and longitudinal research questions, with the acute analysis including the relevant baseline and posttreatment measurements and the longitudinal analysis comparing baseline with the 8-week postintervention assessment.

Given the pilot nature of the study, these analyses will be considered exploratory and will focus primarily on estimating preliminary treatment effects and their uncertainty rather than on formal hypothesis testing. Treatment effects will be expressed as estimated mean differences with corresponding 95% CIs. No formal adjustments for multiple comparisons for the exploratory clinical outcomes will be made. LMMs will use all available data under the assumption that missing data are missing at random. Estimates of outcome variability, including SDs and within-participant correlations, will be used to inform sample size calculations for a future definitive RCT.

The recorded interviews will be transcribed anonymously, and no personal data will be linked to the anonymized transcripts. The transcribed interviews will be analyzed using a phenomenographic approach to identify and describe the qualitatively different ways in which the phenomenon under study is experienced, conceptualized, perceived, and understood [61]. The steps of the phenomenographic analysis are visualized in Figure 2.

**Figure 2.**
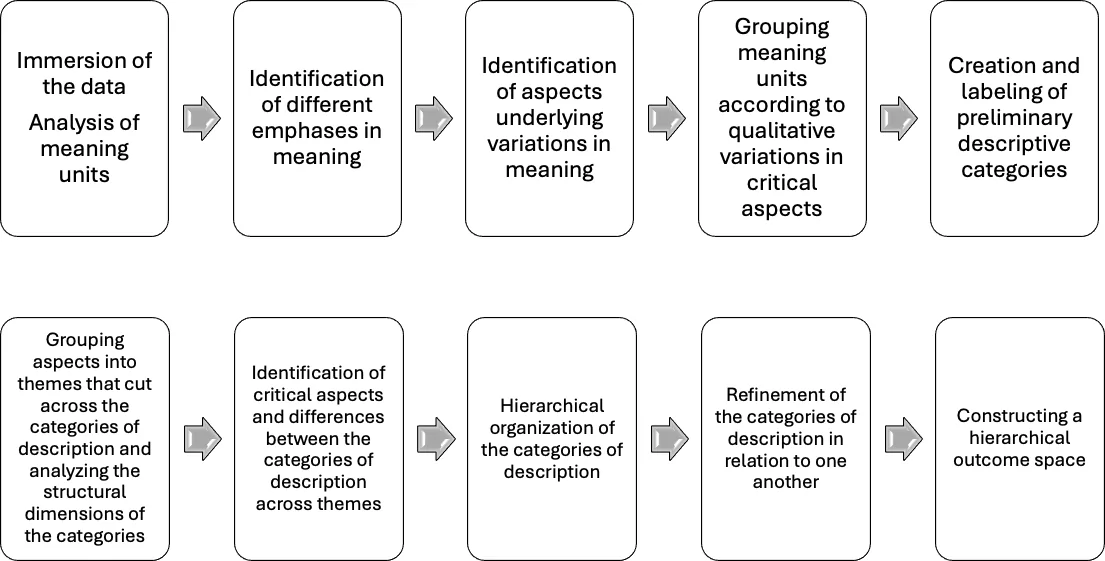
Steps of phenomenographic analysis [61].

Although the participants constitute a small and purposively selected sample, variation in age, sex, and professional experience will be assumed to provide diverse perspectives and contribute to a broader understanding of the phenomenon under study. To enhance credibility, the most relevant meaning units will be carefully selected during the analysis process. Furthermore, quotations from participants will be used to illustrate similarities and differences within the data and to support the interpretation of the findings. If data collection is conducted over an extended period, there will be a potential risk of inconsistency, which could negatively affect dependability [62]. To minimize this risk, the interview guide will be used consistently throughout the interview. The semistructured interview protocols are shown in the appendices (Multimedia Appendices 1 and 2); all the interviews will be conducted by the same researcher, who will also have regular discussions with the research team to ensure a shared understanding of the study procedures and emerging findings. Data analysis will be performed independently by 2 researchers, who will subsequently compare and discuss their findings to reach consensus. A third researcher will be consulted when necessary to resolve discrepancies. Although transferability is inherently limited due to the small sample size, a rich and transparent description of the research context, participants, sampling procedures, data collection, and data analysis will be provided to enhance the overall trustworthiness of the study and allow readers to judge the potential transferability of the findings.

### Ethical Considerations

This study has been designed and will be conducted in strict accordance with the ethical principles outlined in the Declaration of Helsinki [63], as well as national legislation and regulations in Finland, including the Finnish Constitution (731/1999, Sections 6‐23) [64], the Medical Research Act (488/1999) [65], and the Data Protection Act (1050/2018) [66]. As the study involves minors, additional safeguards have been implemented to protect the participants’ welfare, autonomy, and rights. Written informed consent will be obtained from guardians, and assent will be sought from the children themselves. Information about the study will be presented in age-appropriate language to ensure comprehension.

All study-related data will be handled in accordance with the Finnish Data Protection Act (1050/2018), the EU General Data Protection Regulation (GDPR, 2016/679) [67], and institutional data security policies. Study data will be stored securely at the New Children’s Hospital, Helsinki University Hospital (HUS/ULS). Physical materials (eg, consent forms and paper questionnaires) will be stored in locked cabinets in restricted-access areas. Digital data will be stored on secure, HUS-approved platforms that meet institutional and national information security standards.

To ensure participant confidentiality, all research forms, reports, and datasets will be coded with unique study IDs. Personally identifiable information (eg, names and personal identity codes) will be stored separately from the research data and linked only via coded identifiers. Local databases will be password-protected, and access will be limited to authorized study personnel. Interview data collected during the qualitative portion of the study will be fully anonymized prior to analysis, ensuring that individual participants cannot be identified.

All research data will be retained for 15 years following the end of the study, in line with the principles of good clinical practice and institutional guidelines. After the retention period, the data will be securely destroyed, except for anonymized interview material, which will be archived without any identifiable information for potential future research use.

Ethical approval has been obtained from the Regional Ethics Committee of the Hospital District of Helsinki and Uusimaa, which has assessed and approved the protocol (statement ID: HUS/8556/2023). Institutional permission has been granted by HUS’s New Children’s Hospital (research permit ID: HUS/564/2024). The trial is registered in the ClinicalTrials.gov database (trial ID: NCT06837025; accessed on February 20, 2025).

### Patient and Public Involvement

Patients and members of the public were not involved in the design of the research question or study protocol. Participants and their families contribute to the study through their involvement in the intervention and assessment procedures. The outcome measures were selected by the research team with consideration for participant burden and feasibility in children with CP. Once the trial has been published, patients will be informed of the results in a study newsletter suitable for a nonspecialist audience. The results of the study will be submitted for open-access publication.

### Risk Management and Intervention Safety

Participation in the study is voluntary. Written informed consent will be obtained from all guardians or legal representatives of participants below 15 years of age. In addition, assent will be sought from the children themselves, and the researchers will respect the children’s right to refuse or withdraw from the study at any time, without giving any reasons and regardless of parental consent. Information about the study will be presented in age-appropriate language to ensure comprehension. The researchers will adhere to ethical principles that prioritize the autonomy, best interests, and welfare of the participating children.

Participation does not entail any costs for the families and will not interfere with the participants’ regular physiotherapy, which will continue as scheduled. The intervention, either FM or supervised passive stretching, is delivered once weekly and is considered safe and noninvasive.

However, all potential AEs will be actively monitored throughout the study. AEs will be defined as any unfavorable or unintended sign, symptom, or disease temporally associated with the intervention, whether causally related or not. Data on harms will be collected through direct observation, participant or family reports, and standardized AE reporting forms completed by physiotherapists at each intervention session. If any AEs are identified during the study period, these will be reported to the ethics committee within 15 days (approximately 2 wk) and transparently included in the study’s final reporting. SAEs will be reported to the principal investigator within 24 hours and submitted to the ethics committee within 15 days. A summary of AEs and SAEs will be included in any resulting publications. No data safety monitoring board has been and will be established due to the nonpharmacological and low-risk nature of the intervention; however, safety oversight will be managed by the study steering group.

### Dissemination Plan

The results of this study will be submitted for publication in peer-reviewed scientific journals and presented at relevant academic conferences. Reporting will follow the SPIRIT (Standard Protocol Items: Recommendations for Interventional Trials) 2025 statement (Checklist 1) [68] and any applicable extensions, ensuring clarity, transparency, and reproducibility.

## Results

The recruitment began after receiving ethical approval on October 4, 2024, and will continue until we reach a sample of 20 children by the end of 2026. The interventions will start as soon as consent to participate is given. Collecting and analyzing data will begin once the final measurements and interviews have been completed. The research is estimated to be completed by the end of 2027.

## Discussion

FM is increasingly used in the rehabilitation of movement difficulties in children with CP. However, evidence supporting the efficacy of FM remains limited. This pilot RCT was designed primarily to evaluate the feasibility and acceptability of conducting a future definitive RCT comparing FM with traditional stretching in children with CP.

The exploratory clinical outcomes offer initial insights into the potential effects of FM compared with traditional stretching. The qualitative findings complement the quantitative results by providing information on the acceptability and perceived value of the interventions from the perspectives of parents and their children’s regular physiotherapists. These findings contribute to understanding the feasibility of implementing the intervention in routine clinical practice. The findings will also provide important information regarding recruitment, participant retention, intervention delivery, adherence, outcome assessment procedures, and data collection, all of which are essential for future RCTs. To our knowledge, only a few studies have been published that directly examine the effect of an FM intervention on walking in children with CP. The study is expected to provide preliminary information that may help guide future research and the development of rehabilitation strategies for children with CP.

A strength of the study is that it evaluates comprehensive mechanistic outcomes and that both interventions will be performed by 1 physiotherapist, increasing their precision and effectiveness. The FM intervention will be performed by a physiotherapist with experience in both pediatric CP rehabilitation and Stecco’s FM technique. A limitation of this study is the lack of follow-up on other therapies and training during the intervention. Simultaneously prescribed exercises and the use of proper orthotics could significantly influence treatment outcomes. Having 2 intervention groups without a control group may be a further limitation of the study. Finally, due to the nature of the interventions, it was not possible to blind the participants or the study personnel conducting the 6MWT, gait analysis, dynamometry, and clinical assessments.

## Supplementary material

10.2196/92401 Multimedia Appendix 1 Interview guide for caregivers.

10.2196/92401 Multimedia Appendix 2 Interview guide for physiotherapists.

10.2196/92401 Checklist 1 SPIRIT checklist.

## Abbreviations

6MWT: 6-minute walk test
AE: adverse event
CP: cerebral palsy
ECM: extracellular matrix
FM: fascial manipulation
GDI: Gait Deviation Index
GDPR: General Data Protection Regulation
GMFCS: Gross Motor Function Classification Scale
GMFCS-ER: Gross Motor Function Classification Scale-Extended and Revised
HA: hyaluronic acid
HUH/ULS: Helsinki University Hospital New Children’s Hospital
LMM: linear mixed-effects model
MTS: Modified Tardieu Scale
RCT: randomized controlled trial
ROM: range of motion
SAE: serious adverse event
SCALE: Selective Control Assessment of the Lower Extremity
SPIRIT: Standard Protocol Items: Recommendations for Interventional Trials
