# Supplementary material for: Fascial Manipulation Compared to Stretching for Rehabilitation of Children With Cerebral Palsy: Protocol for a Pilot Randomized Controlled Trial
Source: JMIR Res Protoc. 2026 Sep 30;15:e92401. doi: 10.2196/92401 (PMC13626396; doi:10.2196/92401)
Supplement: Supplementary file 1 [file resprot-v15-e92401-s001.pdf]

Semi-structured interview guide for caregivers (fascial manipulation or stretching, post-intervention)

1. Overall experience

- How would you describe your and your child's overall experience of participating in the intervention(s) (stretching and/or fascial manipulation)?
- Did these interventions differ from the rehabilitation your child usually receives? If yes, how?

2. Immediate and longer-term effects

- How did your child feel after an individual session:
  - stretching
  - fascial manipulation
- How did your child feel after completing the entire intervention period?

3. Child's perspective

- What has your child told you about their experiences of:
  - stretching
  - fascial manipulation

4. Everyday functioning

- How is your child managing in daily life, such as at home and at school?
- In your view, how have the interventions affected your child's ability to cope in everyday situations at home and at school?

5. Impact on functioning and participation

- How have the interventions affected your child's:
  - daily functioning
  - physical abilities
  - independence
  - social relationships (e.g., with friends and family)?
- What positive changes have you observed?
- Have you noticed any negative or unintended effects?

6. Dose-related effects (additional therapy)

- Has the additional therapy (increase from once per week to twice per week) affected:
    - your child's daily life?
    - your child's independence?
- How did the additional weekly stretching session influence the physiotherapy provided by your child's regular physiotherapist?
    - If applicable, in what way?
- Overall, was the additional therapy perceived as:
    - beneficial
    - or negative / unnecessary?

7. Perceived effectiveness

- Using a scale from 1 to 5 (1 = no benefit, 5 = very large benefit), how would you rate the overall benefit of:
    - stretching
    - fascial manipulation

8. Added value and potential disadvantages

- What added value, if any, do these interventions provide compared to other rehabilitation approaches?
- Were there any disadvantages (e.g., pain, fatigue, or other concerns)?

9. Final remarks

- Is there anything else you would like to add or highlight that has not been covered?
