# Supplementary material for: Fascial Manipulation Compared to Stretching for Rehabilitation of Children With Cerebral Palsy: Protocol for a Pilot Randomized Controlled Trial
Source: JMIR Res Protoc. 2026 Sep 30;15:e92401. doi: 10.2196/92401 (PMC13626396; doi:10.2196/92401)
Supplement: Supplementary file 2 [file resprot-v15-e92401-s002.pdf]

Semi-structured interview guide for physiotherapists (post-intervention)

1. Background and therapy characteristics

- How long have you been providing physiotherapy to this child?
- How long have you been working with children with cerebral palsy (CP) in general?
- How frequently do you provide physiotherapy to this child?
- What is the typical duration of one therapy session?
- Where is the therapy usually delivered (e.g., at home, clinic, or elsewhere)?

2. Usual physiotherapy practice

- What types of therapy approaches do you typically use in the rehabilitation of children with CP?
- What does usual physiotherapy consist of for this child?

3. Training and expertise in fascial manipulation

- Have you received training in the Stecco method? If yes, what is your level of training?
- How familiar are you with fascial manipulation as a method?
- Do you apply fascial manipulation in your clinical practice?
- In your opinion, how does fascial manipulation differ from conventional physiotherapy for children with CP?

4. Experiences of the interventions (stretching and fascial manipulation)

- What has the child reported regarding their experiences or sensations related to:
    - the stretching intervention
    - fascial manipulation
- What outcomes have you observed following:
    - stretching
    - fascial manipulation
- What positive changes have you noticed?
- Have you observed any negative or unintended effects?
- Using a scale from 1 to 5 (1 = no effect, 5 = very large effect), how would you rate the impact of:
    - stretching

    - fascial manipulation
      on the child's functioning?

5. Impact of additional therapy

- Has the additional therapy (increase from once per week to twice per week) affected:
    - the child's daily life?
    - the child's independence?
- How did the additional weekly stretching session influence the physiotherapy you provided?
    - If applicable, in what way?
- Overall, was the additional therapy perceived as:
    - beneficial
    - or negative / unnecessary?

6. Broader clinical observations

- What other observations have you made regarding the child's functioning during rehabilitation?
- Which other therapeutic approaches do you consider beneficial in the rehabilitation of children with CP?

7. Final remarks

- Is there anything else you would like to add?
- What factors do you consider important to take into account in the rehabilitation of children with CP?
